# Survival of SARS-CoV-2 and bovine coronavirus on common surfaces of living environments

**DOI:** 10.1038/s41598-022-14552-9

**Published:** 2022-06-23

**Authors:** Maiko Watanabe, Takahiro Ohnishi, Sakura Arai, Tsuyoshi Kawakami, Katsuhiko Hayashi, Kenji Ohya, Shouhei Hirose, Tomoya Yoshinari, Satoshi Taharaguchi, Hirohisa Mekata, Takahide Taniguchi, Yoshiaki Ikarashi, Masamitsu Honma, Yukihiro Goda, Yukiko Hara-Kudo

**Affiliations:** 1 Division of Microbiology, National Institute of Health Sciences, Kanagawa, 210-9501 Japan; 2 Division of Environmental Chemistry, National Institute of Health Sciences, Kanagawa, 210-9501 Japan; 3 Laboratory of Microbiology, Department of Veterinary Medicine, Azabu University, Kanagawa, 252-5201 Japan; 4 Center for Animal Disease Control, University of Miyazaki, Miyazaki, 889-2192 Japan; 5 Division of Animal Life Science, Institute of Agriculture, Tokyo University of Agriculture and Technology, Tokyo, 183-8509 Japan; 6 National Institute of Health Sciences, Kanagawa, 210-9501 Japan

**Keywords:** Microbiology techniques, SARS-CoV-2, Viral transmission

## Abstract

Aerosols or saliva containing severe acute respiratory syndrome coronavirus 2 (SARS-CoV-2) can contaminate living environments, and viruses can be indirectly transmitted. To understand the survival potential of the virus, the viral titers of bovine coronavirus (BCoV), as a model virus, and SARS-CoV-2 were measured on porous and non-porous surfaces. The amount of infectious BCoV recovered remained relatively high on non-porous substrates. However, it quickly decreased on several non-porous surfaces such as nitrile rubber. The time taken to reach the limit of detection on non-woven masks, as a porous substrate, was longer than that of non-porous substrates. On porous substrates other than non-woven masks, the amount of virus recovered quickly decreased, and then remained at a low level. Representative substrates were tested with SARS-CoV-2. The decrease in the amount of infectious virus recovered was similar to that of BCoV, although that of SARS-CoV-2 was more rapid. RNA derived from SARS-CoV-2 was also detected using real-time PCR, and it remained on surfaces much longer than infectious virus, on all substrates. Therefore, it is important to measure the viral titer to avoid the overestimation of infectious virus contamination in the environments. Our results suggest that the surface structure was not directly related to viral survivability.

## Introduction

With viral respiratory infections, transmission of the virus often occurs through direct transmission. Direct transmission requires respiratory droplets and aerosols generated by coughs, sneezes, and conversation. It is also believed that respiratory viruses can be indirectly transmitted^1^, by virus deposited on the surface of various materials. In the case of COVID-19, surveys of hospitals and cruise ship environments have revealed the presence of severe acute respiratory syndrome coronavirus 2 (SARS-CoV-2) on various surfaces with which patients came into contact, such as door knobs, TV remote controls, phones, floors, and bedding^2,3^. SARS-CoV-2 particles on the surfaces of various materials may therefore be a source of infection, a hypothesis which has been supported by several epidemiological studies^4,5^. The amount of virus on a surface and the length of time for which viruses survive on materials are crucial for indirect transmission.

Various factors, such as relative humidity, temperature, and the surface properties of a material, affect the amount of infectious virus which can be recovered from a surface. The recovery of infectious SARS-CoV-2 from the surfaces of various materials has been studied by several groups^6–10^, with differing results. These differences may be attributable to differences in the experimental conditions. The relative humidity and temperature influence the evaporation of fluids containing virus particles. With respect to surface structure or roughness, previous researchers have used the terms “porous” or “non-porous” to refer to the surfaces of materials, indicating a rough and coarse, or smooth texture of the surface, respectively. Because porous materials, such as paper and non-woven masks, contain multiple grooves and spaces, they absorb fluid, and trap substances in the fluid. In contrast, non-porous material cannot absorb fluid. The surface properties of a material therefore greatly influence the amount of virus which can be recovered from a surface. Although previous studies used the same materials, including plastic and glass, the authors did not describe the details of the substrates, such as the kinds of plastics, metals, and wood, or the characteristics of the surfaces of these substrates. Unless studies use materials with similar characteristics, the results will vary. Previous studies have mainly investigated non-porous materials, and there are few data pertaining to porous materials. However, individuals constantly come into contact with porous materials such as masks, bank notes, wood, clothing, and paper in the course of daily life. Other factors may be involved in viral recovery from substrates, in addition to surface structure or roughness.

In previous studies, SARS-CoV-2 was detected either by infecting cultured cells or by demonstrating the presence of viral RNA. The infection of cultured cells directly demonstrates the presence of infectious virus, but the detection of RNA reflects the presence of both infectious and non-infectious virus. Therefore, there can be discrepancies between the recovery time of infectious virus as measured by infectivity and that measured by the presence of viral RNA.

To address these problems, we prepared various substrates of known origin, and measured their surface roughness. We detected the virus using both infection of cultured cells and the presence of viral RNA. Experiments with SARS-CoV-2 should be performed in a biosafety level (BSL) 3 laboratory. To facilitate the investigation of a wide range of different surfaces, we initially used bovine coronavirus (BCoV) handled in a BSL2 laboratory as a substitute for SARS-CoV-2. This virus belongs to the genus *Betacoronavirus*, as does SARS-CoV-2, reported by previous studies based on the molecular phylogenetic analysis that BCoV is one of the species most closely related to SARS-CoV-2^11^. After classifying the surfaces into several categories, we investigated the survivability of SARS-CoV-2 on the surfaces of representative characterized materials.

## Results

### Surface roughness of substrates

**Figure 1:**
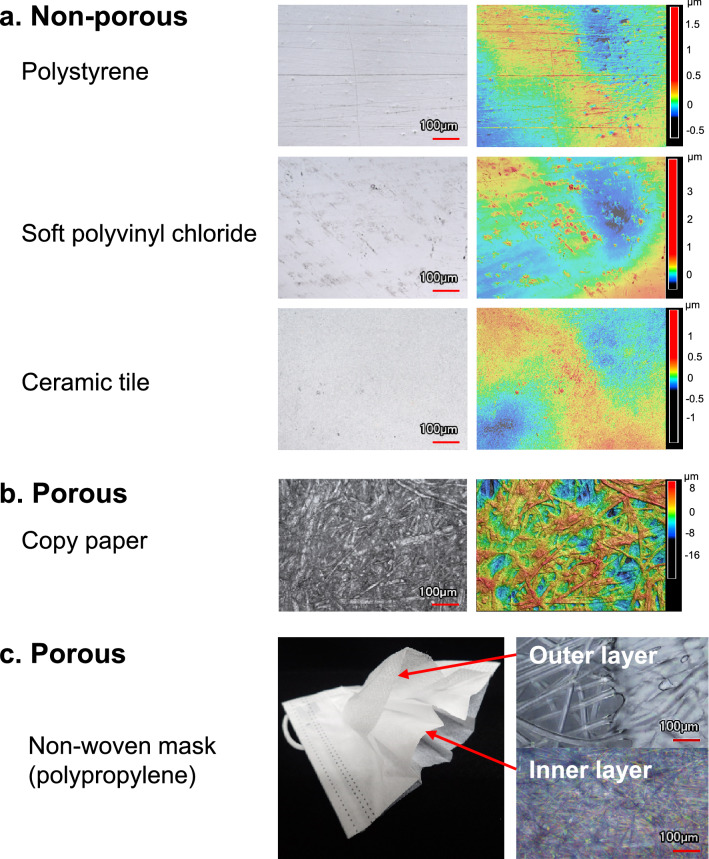
Microscopic observation and 3D images of the surface of substrates (× 20 magnification) investigated in this study. Panel (**a**) and (**b**), left: microscopic observation; right: 3D images on surfaces of representatives of non-porous and porous substrates, respectively, investigated in this study. Panel (**c**), left: appearance and cross-sectional structure of the non-woven mask, right: microscopic observation of two layers of the non-woven mask. The 3D image of the non-woven mask could not be taken because its structure was too deep.

The area surface roughness of each representative substrate was measured, and shown as 3D microscope images (Fig. 1). The microscopic observations showed significant structural differences in 3D images between non-porous and porous substrates at the 100 μm scale. The area surface roughness (Sa; measured in µm) expresses, as an absolute value, the difference in the height of each point compared to the arithmetical mean of the surface. These values are shown in Table 1. The values of Sa on non-porous substrates were 0.040 for float glass, 0.087 for acrylic resin, 0.101 for polypropylene, 0.102 for polystyrene, 0.113 for brass and low-density polyethylene, 0.131 for ceramic tile, 0.150 for soft polyvinyl chloride, 0.167 for stainless steel, 0.295 for melamine resin, and 0.458 for nitrile rubber. The values of Sa on porous substrates were 3.34 for copy paper, 8.19 for polyester cloth, and 12.0 for lauan veneer. Sa was not structurally measurable for the non-woven mask.

**Table 1:** Substrates and the area surface roughness (Sa).

Surface structure	Substrate	Sa^a^ ± stdv
		(μm, n = 8)
Non-porous	Float glass	0.040 ± 0.002
	Acrylic resin	0.087 ± 0.001
	Polypropylene	0.101 ± 0.001
	Polystyrene	0.102 ± 0.003
	Brass C2801 (buff polishing)	0.113 ± 0.003
	Low-density polyethylene	0.113 ± 0.009
	Ceramic tile	0.131 ± 0.020
	Soft polyvinyl chloride	0.150 ± 0.009
	Stainless steel SUS430 (buff polishing)	0.167 ± 0.012
	Melamine resin	0.295 ± 0.043
	Nitrile rubber	0.458 ± 0.042
Porous	Copy paper	3.34^b^ ± 0.30
	Polyester cloth	8.19^b^ ± 0.17
	Lauan veneer	12.0^b^ ± 6.1
	Non-woven mask (polypropylene)	–

^a^× 20 magnification.

^b^Reference value.

### Recovery of infectious virus from surfaces inoculated with BCoV

**Figure 2:**
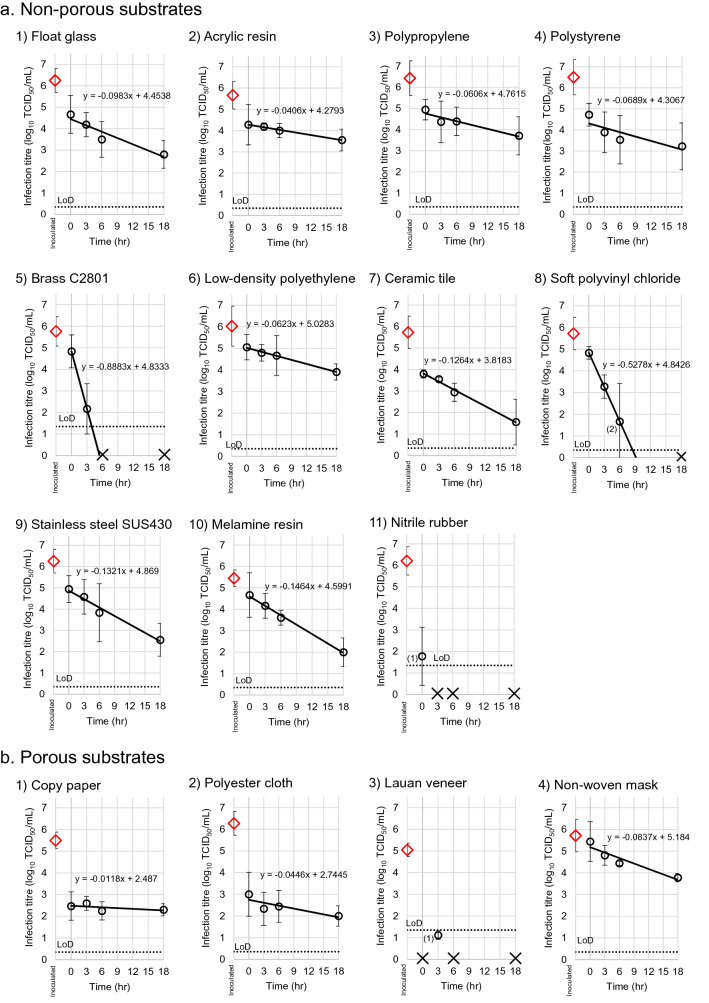
Recovery of infectious virus of BCoV for all surfaces of substrates. The infectious titers of BCoV on surfaces of substrates were measured as TCID_50_/mL in triplicate at 0, 3, 6, and 18 h after inoculation, using HRT-18G cell culture in 96-well plates. Samples at 0 h were measured immediately after drying. Panels A and B show non-porous and porous substrates, respectively. The rhombuses indicate the mean titers of the inoculated virus. The circles indicate the mean titers of the measured infectious titers. The cross marks indicate no-detection (ND) in in triplicate. During assays, A-5) brass, A-11) nitrile rubber, and B-3) lauan veneer showed cytotoxicity, in which collected virus killed HRT-18G cells without virus when using undiluted collections. When calculating a mean value, ND was regarded as 0 TCID_50_/mL for non-cytotoxic materials, and 1 log_10_ TCID_50_/mL for cytotoxic materials. The LoD and the dotted lines indicate the limit of detection of the assays (LoD) at 0.4 log_10_ TCID_50_/mL for non-cytotoxic materials, and 1.4 log_10_ TCID_50_/mL for cytotoxic materials. The numbers in parentheses beside the marks including the data below LoD indicate the number of times above LoD in triplicate. The bars indicate the standard deviations of inoculated or collected virus. The lines in bold indicate the declining rate of virus, calculated using linear regression.

First, the recovery of virus from substrates was measured using the infectious titer of BCoV as a substitute for SARS-CoV-2. The recovery of infectious virus from surfaces inoculated with BCoV is shown in Fig. 2. The limit of detection (LoD) in the virus titer assay was determined to be 0.4 log_10_ TCID_50_/mL. Because of the cytotoxicity of the highest dilution of virus recovered from liquid from brass, nitrile rubber, and lauan veneer, the LoD was determined to be 1.4 log_10_ TCID_50_/mL. Using the slopes of the line of best fit, as shown in Fig. 2, the time to LoD was calculated at a detection limit of 1.0 log_10_ TCID_50_ as an indicator of viral infectivity, assuming a linear decrease in viral recovery based on similar studies^7,12^ for all substrates (Table 2). For non-porous substrates, the times to LoD were 35 h for float glass, 81 h for acrylic resin, 62 h for polypropylene, 48 h for polystyrene, 4 h for brass, 65 h for low-density polyethylene, 22 h for ceramic tile, 7 h for soft polyvinyl chloride, 29 h for stainless steel, and 25 h for melamine resin. The time to LoD for nitrile rubber was not calculated, because its line of best fit could not be drawn without virus titers at more than two points. For non-porous substrates, the times to LoD were 126 h for copy paper, 39 h for polyester cloth, and 50 h for the non-woven mask. The time to LoD for lauan veneer could not be calculated because its line of best fit could not be drawn without virus titers at more than two points. The substrates tested were classified into five groups according to the degree of recovery of the infectious viruses on the surface: (1) float glass, acrylic resin, polypropylene, polystyrene, and low-density polyethylene with high recoveries of infectious virus on non-porous surfaces; (2) ceramic tile, stainless steel, and melamine resin with moderate recoveries of infectious virus on non-porous surfaces; (3) brass, soft polyvinyl chloride, and nitrile rubber with low recoveries of infectious virus on non-porous surfaces; (4) non-woven masks with high recoveries of infectious virus on porous surfaces; and (5) copy paper, polyester cloth, and lauan veneer with low recoveries of infectious virus on porous surfaces.

**Table 2:** Comparison among time to limit of detection for BCoV and SARS-Cov-2.

Surface structure	Substrate	Time to limit of detection (h)			Slope of the line of best fit			Difference of virus titer/RNA copies between time points at inoculation and at 0 h (log_10_ TCID_50_/mL or log_10_ RNA copies/mL)		
		Titer of BCoV^a^	Tier of SARS-CoV-2^a^	RNA copies of SARS-CoV-2^b^	Titer of BCoV	Titer of SARS-CoV-2	RNA copies of SARS-CoV-2	Titer of BCoV	Titer of SARS-CoV-2	RNA copies of SARS-CoV-2
Non-porous	Float glass	35	NT^c^	NT	− 0.0983	NT	NT	1.6	NT	NT
	Acrylic resin	81	NT	NT	− 0.0406	NT	NT	1.4	NT	NT
	Polypropylene	62	NT	NT	− 0.0606	NT	NT	1.5	NT	NT
	Polystyrene	48	18	145	− 0.0689	− 0.1360	− 0.0226	1.8	0.6	0.2
	Brass C2801 (buff polishing)	4	NT	NT	− 0.8883	NT	NT	1.0	NT	NT
	Low-density polyethylene	65	NT	NT	− 0.0623	NT	NT	1.0	NT	NT
	Ceramic tile	22	11	78	− 0.1264	− 0.1852	− 0.0415	1.9	0.8	0.2
	Soft polyvinyl chloride	7	3	36	− 0.5278	− 0.7780	− 0.0905	0.9	0.2	0.2
	Stainless steel SUS430 (buff polishing)	29	NT	NT	− 0.1321	NT	NT	1.3	NT	NT
	Melamine resin	25	NT	NT	− 0.1464	NT	NT	0.8	NT	NT
	Nitrile rubber	NC^d^	NT	NT	NC	NT	NT	4.4	NT	NT
Porous	Copy paper	126	NA^e^	64	− 0.0118	0.0086	− 0.0126	3.0	3.7	1.9
	Polyester cloth	39	NT	NT	− 0.0446	NT	NT	3.3	NT	NT
	Lauan veneer	NC	NT	NT	NC	NT	NT	3.7f.	NT	NT
	Non-woven mask	50	19	129	− 0.0837	− 0.1409	− 0.0260	1.3	0.4	0.0

^a^Calculation with a detection limit of 1.0 log_10_ TCID_50_ for all substrates.

^b^Calculation with a detection limit of 4.8 log_10_ RNA copies/mL for all substrates.

^c^Not tested.

^d^The line of best fit cannot be drawn because virus titers were detected at no more than two points.

^e^The line of best fit was not applicable due to the upward slope.

^f^Calculation with a detection limit of 1.4 log_10_ TCID_50_/mL.

### Recovery of infectious virus on surfaces inoculated with SARS-CoV-2

**Figure 3:**
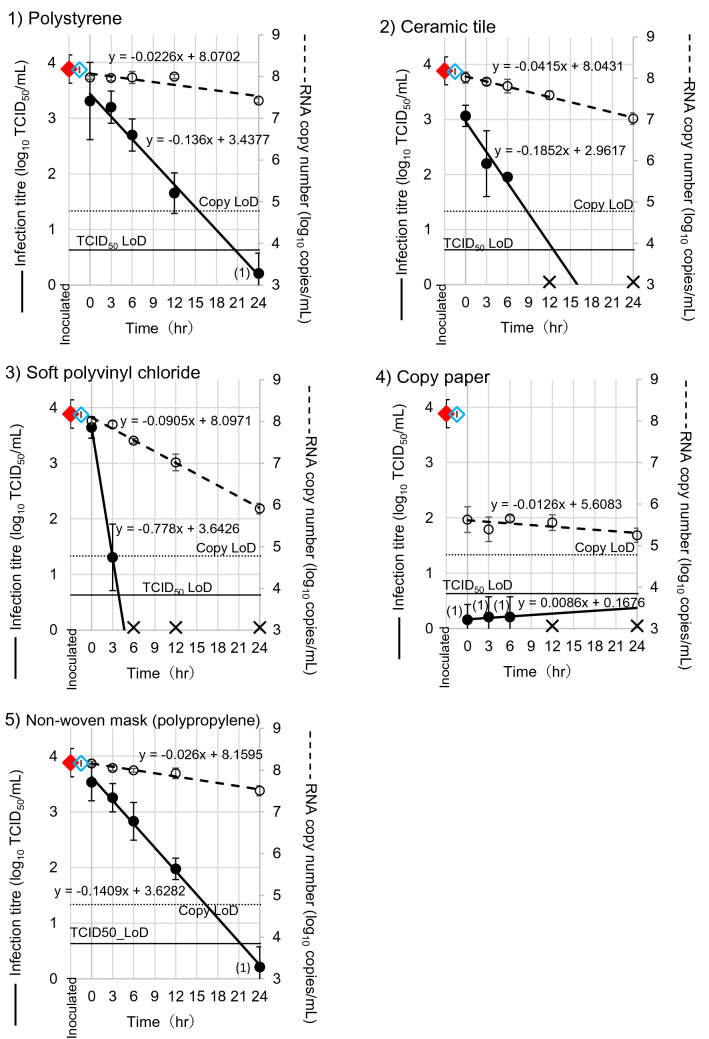
Recovery of infectious SARS-CoV-2 virus from all surfaces of substrates. The infectious titers of SARS-CoV-2 were measured as TCID_50_/mL in triplicate at 0, 3, 6, 12, and 24 h after inoculation, using VeroE6/TMPRSS2 cell cultures in 96-well plates. Samples at 0 h were measured immediately after drying. The left and right y-axes indicate infectious titer (log_10_ TCID_50_/mL) and RNA copy number (log_10_ RNA copies/mL) of the virus, respectively. The closed red and open blue rhombuses indicate the mean titers and RNA copy number of the inoculated virus, respectively. The closed and open circles indicate the mean titers of the measured infectious titers and mean RNA copy numbers of virus, respectively. The cross marks indicate no-detection (ND) in triplicate. When calculating a mean value, ND was regarded as 0 TCID_50_/mL. Horizontal thin and dotted lines mean limit of detection of the assays (LoD) at 0.6 log_10_ TCID_50_/mL for virus titer and 4.8 log_10_ RNA copies/mL for RT-PCR, respectively. The numbers in parentheses beside the points including the data below LoD indicate the number of times above LoD in triplicate. The bars indicate the standard deviations of inoculated or collected virus. The break lines and thick lines indicate the declining rate of virus, calculated using linear regression.

One substrate was selected from each of the five groups of substrates tested, classified by the degree of BCoV viability: polystyrene, ceramic tile, soft polyvinyl chloride, copy paper, and non-woven mask, and used for further experiments with SARS-CoV-2. The recoveries of infectious virus from the surfaces inoculated with SARS-CoV-2 are shown in Fig. 3. From the slopes of the lines of best fit in Fig. 3, the time to LoD was calculated with a detection limit of 1.0 log_10_ TCID_50_/mL for all substrates, and these values are shown in Table 2. For non-porous substrates, the times to LoD were 18 h for polystyrene, 11 h for ceramic tile, and 3 h for soft polyvinyl chloride. For non-porous substrates, the time to LoD was 19 h for the non-woven mask. The line of best fit for copy paper was not applicable, due to its upward slope. Our results indicated that on the non-porous surfaces, the virus viabilities were the highest on polyethylene, moderate on ceramic tile, and lowest on soft polyvinyl chloride. On the porous surfaces, the virus viabilities were higher on non-woven mask and lower on copy paper.

### Comparison of recovery of infectious virus between BCoV and SARS-CoV-2

The two viruses had the common characteristics; the virus recovery from only surfaces of copy paper was significantly reduced by about 4 log_10_ TCID_50_/mL at T = 0. Furthermore, we compared the decreasing rates of SARS-CoV-2 and BCoV on polystyrene, ceramic, soft polyvinyl chloride, and non-woven mask using the two-sided paired-samples *t*-test; SARS-CoV-2 and BCoV showed no significance in the comparison of the decreasing rates (*p* = 0.1015).

### Recovery of viral RNA on surfaces inoculated with SARS-CoV-2

The copy numbers of SARS-CoV-2 RNA on the surfaces of each substrate were quantified using real time RT-PCR (Fig. 3). The inoculum contained 8.2 log_10_ RNA copies/mL, and the remaining number of SARS-CoV-2 RNAs diminished over time on all five substrates. The virus copy number was greatly reduced from 8.2 to 5.6 log_10_ RNA copies/ml after 0 h on copy paper. The LoD in this RT-PCR assay was 4.8 log_10_ RNA copies/mL (Table 2), and was reached at 145, 78, 36, 64, and 129 h on polystyrene, ceramic tile, soft polyvinyl chloride, copy paper, and non-woven mask, respectively. RNA residue from SARS-CoV-2 on the surface remained 7–12 times longer than that of infectious titers.

## Discussion

Several researchers have reported that patient-derived pathogenic viruses, such as influenza virus, SARS-CoV, and SARS-CoV-2^1,2,13,14^, adhere to the surfaces of household items in their environment, and infectious viruses can be recovered. Given the possible risk of infection by SARS-CoV-2 adhering to the surfaces of items touched by many people, it is important to quantitatively evaluate the length of time for which infectability remains.

We carried out a comprehensive evaluation, focusing on the recovery of infectious BCoV and SARS-CoV-2 from various surfaces. In Table 3, the recovery is classified into three types. “High recovery and maintenance” means that the recovery of infectious virus from the surface was at a high level at 0 h after drying of the viral inoculum, and that the infectious virus was maintained for a relatively long time. The type “rapidly decreasing” means that the recovery of infectious virus from the surface at 0 h after drying of the viral inoculum was not strongly decreased, but the infectious virus was immediately inactivated, or that the recovery of infectious virus from the surface at 0 h after drying of the viral inoculum was markedly decreased, and the infectious virus was immediately inactivated. The type “maintain at low level” means that the recovery of the infectious virus from the surface was markedly decreased at 0 h after drying of the viral inoculum, and then it was maintained for a very long time at a low concentration.

**Table 3:** Types of trend in recovery of infectious virus on substrates tested.

Type of recoveries of infectious virus	Substrate	Surface structure
High recovery and maintenance^a^	Acrylic resin	Non-porous
	Ceramic tile	Non-porous
	Float glass	Non-porous
	Low-density polyethylene	Non-porous
	Melamine resin	Non-porous
	Non-woven mask (polypropylene)	Porous
	Polypropylene	Non-porous
	Polystyrene	Non-porous
	Stainless steel SUS430 (buff polishing)	Non-porous
Rapidly decreasing^b^	Brass C2801 (buff polishing)	Non-porous
	Lauan veneer	Porous
	Nitrile rubber	Non-porous
	Soft polyvinyl chloride	Non-porous
Maintain at low level^c^	Copy paper	Porous
	Polyester cloth	Porous

^a^The recovery of infectious virus from the surface at 0 h after drying of the viral inoculum was not strongly decreased, and the recovery of infectious virus from the surface was maintained for a relatively long term.

^b^The recovery of infectious virus from the surface at 0 h after drying of the viral inoculum was not strongly decreased, however, the infectious virus immediately died. Alternatively, the recovery of infectious virus from the surface at 0 h after drying of the viral inoculum was strongly decreased, and the infectious virus immediately died.

^c^Although the recovery of infectious virus was strongly decreased at 0 h after drying of the viral inoculum, it was maintained for a very long time at a low concentration.

In this study we investigated the recovery of infectious BCoV from 15 substrates. The Sa of the non-porous substrates measured in this study ranged from 0.040 to 0.458 μm (Table 1). There was no correlation between Sa and the recovery rate of infectious virus, suggesting that the surface roughness did not have a direct effect on virus survivability. The levels of recovery of infectious BCoV after attachment to the surface was different among substrates with similar physical characteristics (Fig. 2, Table 2). For example, among metallic substrates, the decrease in the recovery of infectious virus from brass was significantly larger than that from stainless steel. One possible explanation for this observation is the inactivation of the virus by copper ions^15^. The level of viral recovery from the surfaces of the plastic resin substrates at 0 h after drying of the viral inoculum was relatively high among substrates tested in this study (Table 2; difference of virus titer between the time of inoculation and at 0 h), so the ability of these substrates to decrease virus particles might be relatively low. Nonetheless, the amount of infectious viruses on nitrile rubber and soft polyvinyl chloride decreased more rapidly than other plastic resin substrates. Various additives are used in nitrile rubber and soft polyvinyl chloride. It appears that the solution of these additives such as plasticizers and antioxidant and vulcanization accelerators^16–18^, from these substrates might affect viral survivability. In fact, thiuram-type vulcanization accelerators (e.g., tetramethylthiuram disulfide) are also used as fungicides^19^, and it has also been reported that nitrile rubber has biocidal activity^20^. We therefore evaluated the type of the trend of the recovery of infectious virus on brass, soft polyvinyl chloride and nitrile rubber as “rapidly decreasing” (Table 3).

With respect to porous substrates, excluding the non-woven mask, the differences in viral titers between the time of viral inoculation and at 0 h after drying of the vial inoculum on the surfaces of copy paper, polyester cloth, and lauan veneer were 3.0, 3.3, and 3.7, respectively (Table 2). These differences were much larger than those of non-porous materials, excluding nitrile rubber, and the viral recoveries immediately dropped to a very low level. However, the slopes of the lines of best fit of the change in SARS-CoV-2 and BCoV titers on these porous substrates were larger than those of other substrates, including the non-porous substrates tested in this study. Infectious BCoV on copy paper and polyester cloth was detected even 18 h after drying of the virus inoculation (Fig. 2), and the time to the LoD of BCoV on copy paper was calculated as 126 h (Table 2). The recoveries of infectious virus from these porous substrates were maintained for a long time at a low level. Therefore, we evaluated the type of the trend of recovery of infectious virus on them as “maintain at low level” (Table 3).

In non-woven masks, there were small differences in viral titer between the time of inoculation and 0 h after drying of the vial inoculum (Table 2). This result suggested that the infectious virus on the surface of the non-woven masks was easier to recover than that on other porous substrates, such as polyester cloth, lauan veneer, and copy paper. Viral particles could be not decreased on the non-woven mask, and were more easily recovered than from the other porous substrates. One possible explanation is that the gaps in the weave on the surface were too coarse to absorb the viral inoculum (Fig. 1). The viral particles became uncollectible on copy paper, polyester cloth, and lauan veneer. The 3D structure of the substrate surfaces could affect viral recovery. A 3D image of the non-woven mask could not be taken in this study, because the structure was too deep (Fig. 1). In the future, it will be necessary to examine the relationship between the structure at the macroscopic level rather than the microscopic level and viral activity for each layer in non-woven masks. Another explanation is that polypropylene, the raw material of non-woven masks, is a hydrophobic polymer, whereas paper and wood are hydrophilic substances. The hydrophobic character of polypropylene may have prevented the viral inoculum from soaking into non-woven fabric.

Therefore, these results suggest that surface roughness did not have a direct effect on viral survivability, and the levels of recovery of infectious virus from surfaces varied among substrates with similar physical characteristics. We hope to clarify the physicochemical properties that have the greatest effect on viral recovery, using the same material with different surface textures, surface water repellencies, and additives, in the future.

The time to the LoD with SARS-CoV-2 for the non-woven mask was 19 h. This was the longest time among the five substrates tested in this study, including non-porous substrates such as polystyrene and ceramic tile (Table 2). Therefore, we evaluated the type of the trend of recovery of infectious virus as “high recovery and maintenance” (Table 3). Katsumi et al. showed that the titer of SARS-CoV-2 in nasopharyngeal swabs of patients were occasionally over 6 log_10_ TCID_50_/mL^21,22^. Because reducing 3.53 log_10_ TCID_50_/mL of SARS-CoV-2 on the non-woven mask to the LoD (0.6 log_10_ TCID_50_/mL) took 21.5 h, as calculated from the slope of the line of best fit for the non-woven-mask in this study (Table 2), the decrease of 6 log_10_ TCID_50_/mL virus on the non-woven mask to the detection limit would take 38.3 h. This calculation indicates the possibility that SARS-CoV-2 on non-woven masks used by patients survives for one or 2 days. The need for caution when handling used masks is clear. However, there is no direct relationship between our results and the effectiveness of non-woven masks in preventing exposure to viral particles from the outside air, or in blocking viral release into the air from the patients’ respiratory systems.

van Kampen et al.^21^ reported that the shedding of infectious virus from COVID-19 patients continues up to 20 days post onset of symptoms, with a median duration of 8 days. Therefore there is a risk of adhesion on everyday objects of infectious virus shed from patients, which could be sources of infection by contact transmission. We must be careful to disinfect the surfaces of the substrates classified as “high recovery and maintenance” in our Table 3. The time to LoD for the infectious titer of polystyrene and non-woven mask, the substrates with the highest recovery rate of infectious SARS-CoV-2, was calculated to be 18 or 19 h after drying of the viral inoculum, with a viral titer of about 4 log_10_ TCID_50_/mL (Table 2). If viruses at a higher titer than this are attached to the surfaces, the infectious virus could survive longer. This observation suggests that we should pay attention while disinfecting items made of these substrates.

The discrepancy between the detection of RNA and the recovery of infectious virus particles suggests that the loss of viability of the virus on these substrates was much faster than decrease in amount of RNA. Therefore, the detection of RNA is not adequate as a marker for the presence of infectious virus. It has previously been indicated that viral RNA was detected on various surfaces touched by SARS-CoV-2 patients^3–5,9^. An investigation in a cruise ship reported that viral RNA could be detected 17 days after patients left^3^. However, as we demonstrated in this study, the detection of viral RNA did not guarantee the recovery of infectious virus particles. Our calculation suggests that SARS-CoV-2 on non-woven masks touched by patients may maintain infectivity for only 38.3 h, as discussed above. Therefore, the transmission of SARS-CoV-2 via the surfaces of environmental substrates may be exaggerated by using the detection of RNA as a marker of the virus. The difference between the detection of viral RNA and the recovery of infectious virus particles must be considered in order to understand the environmental transmission of the virus.

This system of experiments to confirm the gradual decrease in the recovery of infectious virus on the surface of test substrates using BCoV was shown to be useful as an alternative to SARS-CoV-2. Experiments using SARS-CoV-2 should be performed in a BSL3 laboratory. However, BCoV can be handled in a BSL2 laboratory, making it possible to perform experiments safely. It is significant that we have established an experimental system using BCoV as a screening method. Because we compared the dynamics of the survival of the two viruses, and our results with BCoV produced the same trend as those with SARS-CoV-2, BCoV was shown to be valuable as a model virus to test for the recovery of infectious SARS-CoV-2 from surfaces.

## Methods

### Viruses and cells

The BCoV CS5 strain isolated from a nasal swab of cattle showing mild respiratory symptoms in Japan^23^ was used in this study. The nasal swab sample was taken by clinical veterinarians in accordance with the guidelines for animal ethics of Miyazaki Agricultural Mutual Relief Association for the diagnostic and therapeutic purpose under the owner’s consent. The veterinarian in University of Miyazaki isolated the viral strain from the swab sample with the approval of Infectious Disease Research Unit Management Committee, the Center for Animal Disease Control. The SARS-CoV-2; 2019-nCoV JPN/TY/WK-521 strain was obtained from the National Institute of Infectious Diseases in Japan.

For the propagation of BCoV, HRT-18G cells were cultured in Dulbecco’s Modified Eagle’s Medium high glucose (DMEM-high glucose; Thermo Fisher Scientific, Waltham, MA, USA) supplemented with 10% FBS (Bovogen Biologicals Pty Ltd., Keilor, Australia) and 1% Antibiotic–Antimycotic (Thermo Fisher Scientific)^23^. The virus and cell cultures were maintained for 5 days at 37 °C in 5% CO_2_, and cell supernatants and lysates were collected. The virus was concentrated and purified by ultracentrifugation using the sucrose density gradient method^24,25^, and was suspended in HPLC grade distilled water (Kanto Chemical Co., Ltd., Tokyo, Japan). Purified BCoV was diluted tenfold in DMEM-high glucose with the supplements described above, and used in inoculation experiments on surfaces.

For the propagation of SARS-CoV-2, VeroE6/TMPRSS2 (JCRB 1819) cells were cultured in Dulbecco’s Modified Eagle’s Medium low glucose (DMEM-low glucose; Thermo Fisher Scientific) supplemented with 10% FBS, 2.5 µg/mL G418 disulfate aqueous (Nacalai tesque, Inc., Kyoto, Japan) and 1% Antibiotic-Antimycotic^7,26^. After 5 days at 37 °C in 5% CO_2_, cell supernatants and virus were collected, and diluted tenfold in DMEM-low glucose without supplements, and used in inoculation experiments on surfaces.

### Substrates

The surfaces of 15 commercially available substrates representing a variety of surfaces in living environments were tested in this study (Supplementary Table S1). These substrates were purchased on the market. A 5 by 5 cm piece of float glass, acrylic resin, polypropylene, polystyrene, brass C2801 (Cu 60%, Zn 40%), low-density polyethylene, ceramic tile, soft polyvinyl chloride, stainless steel SUS430, melamine resin, and nitrile rubber, and of a porous substrate of lauan veneer were used in the inoculation tests. Three 1 by 1 cm pieces of the porous substrates copy paper, polyester cloth, and non-woven mask made of polypropylene (including the entire inner and outer layers) were used in the inoculation tests. The samples were sterilized using ethylene oxide gas. Experiments were performed for each surface in triplicate.

### Surface roughness

The area surface roughness (µm), which expresses, as an absolute value, the difference in height of each point compared to the arithmetical mean of the surface, was measured using a VK-X1000 3D microscope by Keyenece (Osaka, Japan). The imaging field was 705 µm in the X axis and 528 µm in the Y axis (× 20 magnification). Sa was calculated as the average value of the measurements taken at eight non-overlapping observation surfaces on the sample surface (*n* = 8).

### Recovery of virus from surfaces

In the tests with BCoV, virus-inoculation tests were conducted using all 15 substrates (Supplementary Table S1). In the tests with SARS-CoV-2, virus-inoculation tests were conducted using five samples selected with reference to the experimental results of the BCoV; polystyrene, ceramic tile, soft polyvinyl chloride, copy paper, and non-woven mask. The viral inoculation and recovery-test was conducted on these surfaces of substrates referring to previous studies^12,27,28^. A 5 by 5 cm piece of substrate or three 1 by 1 cm pieces of substrate in were placed in a sterile glass petri dish. Sixty microliters of viral inoculum divided into six to twelve spots was loaded on the center of a surface. The Petri dish was opened and allowed to dry in a safety cabinet for about 60 min.

After drying, the substrate was placed in a glass Petri dish in a constant temperature and humidity incubator set at 75% RH and 25 °C (annual average outdoor air humidity in Tokyo, Japan in 2018 and 2019, as published by the Japan Meteorological Agency)^29^. BCoV was recovered at four time-points; time 0 (immediately after drying), and after 3 h, 6 h, and 18 h, in 300 µL drops of DMEM-high glucose supplemented with 1% FBS, 1% Antibiotic–Antimycotic and 2.5 µg/ml pancreatin (Fujifilm Wako Pure Chemical Corporation, Richmond, VA, U.S.A.) by pipetting on the surface for a minute, and collecting as much as possible of the fluid into a microtube. SARS-CoV-2 was recovered at five time-points; time 0 h (immediately after drying), and after 3 h, 6 h, 12 h, and 24 h, in 300 µL drops of DMEM-low glucose supplemented with 1% FBS, 2.5 µg/ml G418 and 1% Antibiotic–Antimycotic by pipetting on the surface for a minute, and collecting as much as possible of the fluid into a microtube. Each recovery of virus was repeated twice on each surface, and a total of 600 µl of inoculum was collected. The negative controls consisted of the same surfaces without prior inoculation of the virus. The infectious virus titer in a recovery collected at each time point was compared, to evaluate the efficiency of infectious virus recovery. This experiment was repeated in triplicate.

### Quantitation of infectious virus titers

The amounts of infectious virus on all surfaces were quantified using end-point titration with a tenfold serial dilution of virus recovery in DMEM with the supplements described above. SARS-CoV-2 was inoculated onto fresh monolayers of cells (Vero E6/TMPRSS2) in a 96 well plate. The culture was maintained for 4 days at 37˚C in 5% CO_2_. BCoV was inoculated onto fresh monolayers of HRT-18G cells in a 96-well plate. The culture was maintained for 6 days at 37˚C in 5% CO_2_. After incubation, the cells were monitored to detect virus-induced cytopathic effect (CPE). The virus titers were calculated using the Reed–Muench method^30^.

### Statistical analysis of viral titers

When the titers were below the limit of quantification of the Reed–Muench method, the titer value was estimated by dividing the numbers of wells in which CPE appeared by the equivalent volumes of undiluted recovering liquid in an assay (e.g. 1 CPE well in 0.22222 mL of equivalent undiluted volume in a viral titer assay leads 0.65 log_10_ TCID_50_/mL). The cytotoxicity was observed at the highest dilution of a virus recovering liquid from three kinds of substrates, such as brass, nitrile rubber, and lauan veneer. Using this estimation, the LoD in the viral titer assay was determined as 0.6 log_10_ TCID_50_/mL for SARS-CoV-2, 0.4 log_10_ TCID_50_/mL for BCoV with non-cytotoxic materials, and 1.4 log_10_ TCID_50_/mL for BCoV with cytotoxic substrates, as described above. An assay without CPE was regarded as ND. For the calculation of the mean values and standard deviations of titers, ND was regarded as 0 TCID_50_/mL. The titer declining lines were calculated by linear regression using the mean titers, excluding all ND data in a time point. For the comparison of decreasing rates, two-sided paired-samples t-test was performed with the significance level at *p* < 0.05.

### Quantitation of SARS-CoV-2 RNA using real time RT-PCR

Viral RNAs were extracted from the SARS-CoV-2 recoveries from surfaces using the QIAamp Viral RNA Mini Kits (Qiagen GmbH, Hilden, Germany) following the manufacturer’s instructions. RT-PCR was performed using One Step PrimeScript RT-PCR Kits with Primer/Probe set N2 (Takara Bio, Shiga, Japan)^31^. The cycling conditions were: reverse transcription for 5 min at 42 °C, initial denaturation for 10 s at 95 °C, then 45 cycles of 5 s at 95 °C and 30 s at 60 °C on an Applied Biosystems 7500 Real-Time PCR System (Thermo Fisher Scientific). To quantify the viral RNA copy numbers, a standard curve was generated using Positive Control RNA Mix (2019-nCoV; Takara Bio). The time to LoD for SARS-CoV-2 was calculated at a detection limit as 4.8 log_10_ RNA copies/mL for all substrates.

## Supplementary Information

Supplementary Information.

## Data Availability

The authors confirm that all data supporting the findings of this study are available within this article and its supplementary material.
