# Supplementary material for: Survival of SARS-CoV-2 and bovine coronavirus on common surfaces of living environments
Source: Sci Rep. 2022 Jun 23;12:10624. doi: 10.1038/s41598-022-14552-9 (PMC9218704; doi:10.1038/s41598-022-14552-9)
Supplement: Supplementary file 1 — Supplementary Information. [file 41598_2022_14552_MOESM1_ESM.docx]

**Supplementary Table S1. Substrates tested in this study**

| Surface structure | Substrate | Manufacture or seller | Product number or  the Japanese Industrial  Standard (JIS) number |
| --- | --- | --- | --- |
| Non-porous | Float glass | AGC Inc. | JIS R3202 |
|  | Acrylic resin | Mitsubishi Rayon Co.,Ltd. | L001 |
|  | Polypropylene | Showa Denko Materials Co.,Ltd. | PP-N-BN |
|  | Polystyrene | Toyo styrene Co., Ltd. | MW1D |
|  | Brass C2801  (buff polishing) | Mitsubishi Materials Corporation | JIS H3100 |
|  | Low-density polyethylene | Showa Denko Materials Co.,Ltd. | EH-N-AN |
|  | Ceramic tile | Hiromi Tobo Co., Ltd. | P-15-105 |
|  | Soft polyvinyl chloride | Yamazol Co., Ltd. | Unknown |
|  | Stainless steel SUS430  (buff polishing) | JFE Steel Corporation | JIS G4304 |
|  | Melamine resin | Daiwa Co., Ltd. | BK5080KLC2 |
|  | Nitrile rubber | Maxell Ltd. | Unknown |
| Porous | Copy paper | Ricoh Co., Ltd. | 901442 |
|  | Polyester cloth | Japanese Standards Association | 670110 |
|  | Lauan veneer | Standard Testpiece Co., Ltd. | Unknown |
|  | Non-woven mask | Maeda Seisakusyo Co., Ltd. | MASK-3 |
